# Navigating barriers: two-year follow up on recommendations to improve the use of maternal health guidelines in Kosovo

**DOI:** 10.1186/s12889-016-3641-5

**Published:** 2016-09-15

**Authors:** Julia E. Moore, Sami Uka, Joshua P. Vogel, Caitlyn Timmings, Shusmita Rashid, A. Metin Gülmezoglu, Sharon E. Straus

**Affiliations:** 1 Li Ka Shing Knowledge Institute, St. Michael’s Hospital, 30 Bond Street, Toronto, ON M5B 1W8 Canada; 2 World Health Organization Country Office, Pristina, Kosovo; 3 Department of Reproductive Health and Research, World Health Organization, UNDP/UNFPA/UNICEF/WHO/World Bank Special Programme of Research, Development and Research Training in Human Reproduction (HRP), Geneva, Switzerland; 4 Department of Medicine, Faculty of Medicine, University of Toronto, 1 King’s College Circle, Medical Sciences Building, Toronto, ON M5S 1A8 Canada

**Keywords:** Guideline implementation, Maternal health, Process evaluation, Realist evaluation, Low and middle income country

## Abstract

**Background:**

Although there are a growing number of initiatives aimed at supporting guideline implementation in resource-constrained settings, few studies assess progress on achieving next steps and goals after the initial activities are completed and the initial funding period has ended. The aim of the current study was to conduct a qualitative process evaluation of progress, barriers, facilitators, and proposed solutions to operationalize nine recommendations to prepare Kosovo to implement the 2012 World Health Organization (WHO) prevention and treatment of postpartum haemorrhage guideline.

**Methods/Design:**

In 2012, we co-created nine recommendations designed to support implementing the WHO’s guideline on the prevention and treatment of postpartum haemorrhage in Kosovo. The current study uses a realist evaluation approach to assess activities and progress two years after the recommendations were developed. The study involved conducting qualitative focus groups and one-on-one interviews with participants from the first meeting to evaluate the activities and progress on the nine recommendations.

**Results:**

Forty-three participants provided insights into the barriers and opportunities experienced to date and proposed future directions. Although progress has been made towards implementation of a number of the recommendations, scaling up has been limited by barriers, such as lack of awareness, limited resources, and evaluation challenges. Participants proposed addressing these barriers by building within- and between-country partnerships to facilitate guideline implementation. In addition, participants reported less progress on implementing recommendations related to broader cultural changes, which indicates a need for specific and actionable recommendations to operationalize implementation efforts.

**Conclusions:**

In the two years since the initial meeting, there has been mixed progress on the recommendations. Based on participant feedback, we refined the recommendations so that they can be operationalized by health care system stakeholders in Kosovo to further support implementation efforts. It is beneficial to share these lessons learned throughout the implementation process to inform next steps in Kosovo and offer ideas for use in other settings.

**Electronic supplementary material:**

The online version of this article (doi:10.1186/s12889-016-3641-5) contains supplementary material, which is available to authorized users.

## Background

Despite the large number of high-quality, evidence-based guidelines available, clinicians do not routinely use guideline recommendations in practice [1]. The goal of knowledge translation (KT) is to address these research-to-practice gaps by using evidence on the best ways to support systemic, organizational, and individual change [2]. There are a growing number of initiatives aimed at supporting guideline implementation in low and middle-income countries, (LMICs) [3, 4], yet very few clearly defined implementation strategies are currently used [5], and even fewer studies follow up on what has been done after the initial funding has expired. Challenges to applying research evidence, and to implementing guidelines in particular, are compounded when working in LMICs, where resources are constrained [6] and there is a history of non-governmental organizations (NGOs) allocating short-term funding without monitoring and evaluating progress or planning for long-term sustainability [7].

Globally, many LMICs struggle with high rates of maternal and perinatal mortality. Although there have been improvements in maternal and infant outcomes, maternal mortality rates (MMR) have declined by 45 % from 1990 to 2013, nevertheless, for every 100 000 live births in 2013, an estimated 210 women died due to complications in pregnancy and childbirth. Moreover, 2013 data showed that the MMR in LMICs was 14 times higher than that in high-income countries [8]. Kosovo, where the current study was conducted, has a perinatal mortality rate (18.7 % in 2011) that is higher than that of other countries in Europe [9]. Furthermore, Kosovo’s perinatal mortality rate increased from 2005 to 2008, even though 95 % of women reportedly gave birth in a health care facility. During the years following this period (2008–2010), the second most common cause of maternal mortality in Kosovo^1^^1^ was postpartum haemorrhage (PPH; 5 out of 24 deaths or 21 %) [10]. Based on these trends, we focused our implementation initiative in Kosovo on the 2012 World Health Organization (WHO) guidelines for the prevention and treatment of PPH [10].

The current study is a partnership between health care system stakeholders working in maternal health in Kosovo, the UNDP/UNFPA/UNICEF/WHO/World Bank Special Programme of Research, Development and Research Training in Human Reproduction (HRP), and the KT Program at St. Michael’s Hospital (SMH) in Toronto, Canada. In 2012, we conducted a survey, two focus groups, a prioritization activity, and a workshop to plan for the implementation of maternal and perinatal health guidelines in Kosovo [11]. Primary outcomes of the workshop included a list of prioritized guideline recommendations, barriers and facilitators to guideline adherence, and nine key recommendations, which served as potential actions to move guideline implementation efforts forward. Although some of the barriers pertained to larger resource and system-level issues (e.g., lack of a standardized data collection system; post-conflict fragmentation of the health care system; and human resource challenges, including high turnover of health care workers), several barriers were more easily turned into actionable recommendations (e.g., develop a guideline implementation working group).

Researchers have found that implementation efforts are not always effective and typically produce small effect sizes [12]. This suggests that it is important to not only ensure that implementation strategies are carefully prioritized, selected, and matched to address known barriers and facilitators, but also that implementation activities are continuously monitored and evaluated. Because implementation is an iterative process, the findings of a process evaluation are critical to the implementation pathway and can inform how an implementation strategy should be revised or discontinued [2]. Process evaluations provide an opportunity to see what progress has been made, change course if necessary, and refocus implementation efforts. They also give program developers and implementers an opportunity to infuse evidence and theory into the implementation process, which further strengthens the quality of the initiative [13, 14].

### Study objectives

The aim of the current study was to conduct a qualitative process evaluation, using a realist evaluation approach [15], of activities and progress on the nine recommendations made to prepare Kosovo to implement the WHO prevention and treatment of PPH guideline. The period of interest for this evaluation was from October 2012 until November 2014. The specific objectives were to: 1) understand progress to date on each 2012 recommendation; 2) identify barriers and facilitators to progress on each recommendation; 3) capture stakeholders’ proposed future directions; and 4) develop refined recommendations to facilitate implementation.

## Methods

Using a realist evaluation approach [15], we conducted qualitative focus groups and one-on-one interviews to evaluate the activities and progress on the nine recommendations that emerged from a 2012 prioritization meeting. The purpose of this process evaluation was to understand progress to date and to support a renewed interest in developing, adapting, and implementing guidelines to change provider behaviour and patient outcomes, focused in maternal health. As a result of these goals, we used a realist evaluation approach [15, 16] to conduct the study and develop actionable recommendations to move forward. Using a realist evaluation approach assumes that implementation is inextricably linked with the context, meaning that the outputs and outcomes of an intervention cannot be separated from the setting in which the intervention was implemented. Since contextual factors change over time, it is imperative to revisit goals and progress to date; this process of refining goals can also serve to reinvigorate stakeholder buy-in, enhance motivation, and identify next steps that are feasible and appropriate at a given juncture in the implementation process.

### Setting

Focus groups and interviews were conducted in Kosovo with support from the WHO Country Office in Pristina, Kosovo; the United Nations Development Programme (UNDP)/United Nations Population Fund (UNFPA)/United Nations Children’s Fund (UNICEF)/WHO/Special Programme of Research, Development and Research Training in Human Reproduction (HRP), Department of Reproductive Health and Research, World Health Organization Headquarters in Geneva, Switzerland; and the KT Program at St. Michael’s Hospital in Toronto, Canada. Focus groups were conducted during an in-person meeting held in Pristina, Kosovo in 2014.

### Participants

Local stakeholders (e.g., policymakers, researchers/academics, frontline health care providers, and representatives from professional associations and non-governmental organizations) who took part in the two-day workshop in 2012 were invited to participate in a focus group. They were told that they would be asked to discuss progress, challenges, and opportunities related to the nine key recommendations made to improve the uptake of the WHO guideline recommendations for the prevention and treatment of PPH. Additionally, five local stakeholders were identified as key informants on maternal health in Kosovo and were invited to participate in one-on-one phone interviews. Participants were identified because they play a key role in guideline development and/or guideline implementation; two of the four participants were also present at the in-person meeting.

### Data collection

Facilitators and interviewers used a semi-structured guide during both the focus group (FG) session (which lasted approximately 60 min) and interviews (which lasted approximately 45–60 min; Additional file 1). Participants received a summary of the 2012 workshop findings and recommendations to review before the FG/interview (Additional file 2). The summary document included a column where participants had the option of anonymously commenting on the progress made on each recommendation. The FG session and one-on-one interviews were audio recorded. The FG session was conducted in Albanian and recordings were simultaneously translated into English and transcribed by a translator. Interviews were conducted in both English and Albanian and were transcribed verbatim or simultaneously translated and transcribed by an independent translator, respectively.

### Data analysis

Two qualitative analysts (not involved with data collection) independently performed a qualitative analysis of the transcripts by using a thematic analysis approach [17]. First, the analysts familiarized themselves with the data to develop coding themes. Second, these themes were refined into categories, which were then compared, sorted, and grouped to develop a *framework of findings.* Third, all transcripts were imported into qualitative analysis software (NVivo 10) and the analysts independently coded using the developed framework. Inter-rater reliability (i.e., the degree of agreement between two coders) was compared by calculating percentage agreement within NVivo 10; any discrepancies (i.e., < 80 % agreement) between the analysts were reconciled through discussion. Using the technique of integration, FG and interview data were considered together to draw meaningful and pertinent recommendations that are feasible and relevant for the Kosovo context. Revised recommendations were developed collaboratively with the study authors, including end-users (Kosovo partners), guideline development experts (WHO partners), and KT experts (SMH partners).

## Results

Thirty-nine stakeholders, (e.g., policymakers, researchers/academics, health care providers, professional associations, non-governmental organizations) took part in the FG, and 17 of the 39 stakeholders completed the summary worksheets. Of the 39 participants, 14 participated in the original 2012 meeting (there were 28 participants at the 2012 meeting); the remaining participants have subsequently been involved with activities related to the recommendations. Table 1 presents information on the professional roles of the FG participants. Four of the five invited stakeholders participated in one-on-one interviews.

**Table 1:** Focus group participant professional role information

Level of health care system	*n =* 39	%
University Clinical Centre	14	35.9
Regional Hospital	13	33.3
Ministry of Health	3	7.7
Professional Association	3	7.7
Family Medicine Centre	2	5.1
City Hospital	2	5.1
International Organization	1	2.6
National Institute of Public Health	1	2.6
Role	*n =* 39	%
Obstetrician	14	35.9
Midwife/Nurse	11	28.2
Director/President/Chief	9	23.1
Family Physician/Other doctor	3	7.7
Other	2	5.1

During the process evaluation conducted in 2014, participants described factors (i.e., barriers and facilitators) they perceived to have influenced progress made regarding the recommendations identified in the original workshop in 2012. For recommendation-specific barriers and facilitators refer to Table 2.

**Table 2:** Summary of progress, barriers, facilitators, and future directions for nine recommendations to support the implementation of WHO guideline on the prevention and treatment of PPH as identified by participants

Recommendation	Progress to date	Barriers	Facilitators	Possible future directions
#1: Create a centralized system for data collection across clinical setting as well as for formal and informal channels for practice sharing.	*Centralized system for data collection* • Although a centralized system has not been developed, preliminary steps to formalize the system are underway. • Piloted efforts to consolidate all institutions in improving data collection processes: o Equipment installed in Prizren Regional Hospital a year ago is not yet functional; o 7 FG discussions have been conducted to facilitate the process for designing relevant software for this system. • Established a health information system using intranet, which is used for data collection at local hospitals/family medicine centres. There is no consistent data reporting process. • Until full implementation of the directives outlined by MOH in the Strategy of Information 2010–2019 is completed, local level data is being collected through different sources (e.g., charts, registers, reports to the National Institute of Public Health). *Practice sharing* • The Mother, Child and Reproductive Health Office, MOH with technical support from UN agencies organized coordination meetings in which all stakeholders involved in maternal and child health gather to discuss progress or barriers and share individual experiences. • Key informant mentioned taking steps to engage in practice sharing through coordination meetings.	*Centralized system for data collection* • No barriers reported by participants. *Practice sharing* • Meetings lack a person with decision-making power. • Lack of buy-in from stakeholders. *Practice sharing* • These meetings were held frequently, but have become less frequent recently. • Stakeholders involved in these meetings do not have any decision-making power, they can only advocate, voice their concerns and recommendations.	*Centralized system for data collection* • Implementing the information strategy by creating a centralized data collection system is a priority of the Secretarial Health Strategy 2014–2020 and is supported by other international projects. *Practice sharing* • Working groups developed to share information are attracting the right people (i.e. MOH, leaders in maternal health).	*Centralized system for data collection* • Local data collection initiatives are perceived to have been effective in yielding usable data (e.g., Annual Health Statistics 2013). • Participants believe that once the centralized system is operationalized and national level data is in electronic format, it will provide an adequate view of mortality and fatality and help select and prioritize topics for guideline development. *Practice sharing* • A key informant suggested that the MOH officially endorse these meetings, and use them as a basis for creating an official body with adequate representation from key institutions and decision-making authority.
#2: Incorporate standards into clinical practice including a monitoring system for guideline adherence.	• Although standards and protocols have been developed, participants perceived that they have not been incorporated into medical centres adequately and comprehensively. • Steps have been taken to encourage health care providers to incorporate standards into practice, including: o Guidelines for primary care have been developed and collected in a resource book shared with family doctors to encourage incorporation of standards in their practice. However, the development and adaptation of these guidelines were not reviewed or approved by the MOH and activities were completed locally, not nationally. o In Prizren Regional Hospital, each ambulance has been equipped with a manual as a convenient reference for staff. • WHO guidelines are used and monitored on a case-by-case basis at institutional and regional levels, based on individual preference. There is no central system for monitoring the utilization of WHO guidelines.	• Lack of awareness of local guidelines. • Lack of clarity regarding the difference between a clinical protocol and guideline. • Lack of an evaluation scale or indicators to evaluate action. • Lack of human resources to consolidate information for a central monitoring system. • Lack of financial resources to incorporate standards into practice.	• Engaging clinicians who have successfully implemented protocols in their own clinics. • Scaling up local auditing to monitor the use of clinical guidelines.	*In progress:* • A new division in the MOH will be created to monitor and evaluate health services, including monitoring clinical protocols and guidelines. • Placing protocols in all clinics to encourage their use. *Possible:* • MOH could validate the use of the Effective Perinatal Care (EPC) programme as the national programme and advocate for its use as a basis for adapting national protocols. • The program could increase the number of inspectors in the MOH and contribute to monitoring and evaluation of implementation, validation and re-validation of health institutions and experts.
#3: Create motivational strategies such as incentives for health care staff (including managers and clinicians) to encourage guideline adherence.	• The majority of participants indicated that no motivation or incentive strategies are currently being used. • Some mentioned modest support of local level incentives, such as motivating trained staff to give seminars/lectures to encourage guideline implementation as part of continuing medical education. • MOH has drafted a directive which outlines job description, competencies and coefficient of salaries to create more incentives for nurses and midwives, but this has not yet been approved. • A national level performance-based incentive program is currently in development.	• Barriers to using incentives for health care staff, such as the Payment for Performance (P4P), were identified: o Causes dissatisfaction among health care workers due to the lack of clear indicators for performance evaluation at individual/ institutional levels; o Perceived as being subjective and biased; o Could result in engaging individuals based on their influence on decision making bodies rather than performance; o Could result in false/over reporting on guideline adherence. • Difficult to expect adherence to internationally developed guidelines (due to unavailability of nationally developed guidelines) without approval/endorsement by the MOH. • Lack of clarity regarding the difference between a clinical protocol and guideline. • Difficult to motivate private practitioners as MOH does not have authority over private practice.	• Presence of a health law (2012) and health insurance law (2014) allows payment based on performance. • Performance-based payment could motivate health care staff to further develop clinical protocols, implement change and improve quality of health care. • Luxembourg government provided a monetary donation to encourage guideline adherence among health care workers. • Participation in maternal and perinatal health care conferences allows for greater involvement in and knowledge of implementing guidelines to address the three most frequent maternal health problems.	*In progress:* • The national level performance-based incentive program will be applied in all institutions as part of the action plan and strategies to strengthen the health sector. *Possible:* • In the future, health care workers will have to be relicensed every 5 years, therefore they could be provided with credits and free days for joining working groups for guideline development and adaptation.
#4: Increase communication across stakeholder groups including clinicians, managers, and policymakers through participation in activities such as guideline development committees.	• Communication between MOH and clinicians organizations has improved over the past year but only occurs during infrequently organized gatherings/meetings. • Although no guideline development committee has been developed, some participants identified stakeholders to include in these committees, but are waiting for approval from MOH to proceed. • Previously established committees have become disengaged due to political circumstances and staffing changes at the MOH; awaiting direction from the MOH to proceed.	• MOH level barriers (e.g., presence of official procedures, formalities, and bureaucracy and lack of decisional authority given to members outside MOH). • Funding for GDG (guideline development group).	• Participation in guideline development meetings provides opportunities to increase communication across stakeholder groups.	• Once established, the development committee can progress with guideline and protocol development.
#5: Create a guideline implementation working group with representative stakeholders at the local level.	• A central level guideline implementation working group has been formed for approval but the group is not yet operational. • The national committee is awaiting administrative instruction to regulate the process of guideline development/adaptation and MOH approval for the guideline development committee. • FG participants explained how the national committee is waiting for requests to come from clinicians in order to establish the guideline implementation working group. • In contrast, interview participants explained how an implementation working group has not been created because they are waiting on MOH approval.	• MOH level barriers (e.g., presence of official procedures, formalities, and bureaucracy and lack of decisional authority given to members outside MOH).	• No facilitators identified by participants.	• Once the guideline development committee is active they can move forward with development of an implementation working group.
#6: Develop a small working group with local representatives from clinician groups, the MOH guidelines committee and quality portfolio, clinical or health services researchers, and the WHO to move forward with implementation.	• A small working group has not been created due to road blocks at the MOH level (i.e., waiting for approval for the development committee).	• MOH level barriers (e.g., presence of official procedures, formalities, and bureaucracy and lack of decisional authority given to members outside MOH).	• No facilitators identified by participants.	• No future direction identified by participants.
#7: Consider offering workshops on guideline development methods, including use of GRADE (Guyatt et al., 2008), on appraisal of guidelines using AGREE, and on guidelines adaptation (National Collaborating Centre for Methods and Tools [NCCMT], 2011), for representatives from the MOH and clinical groups.	• 3 guideline development training sessions have been offered: i. AGREE/ADAPTE Workshop in Kosovo 2013 ii. UNFPA supported workshop Skopje (Macedonia) iii. Suisse Diamonds • Knowledge gained by workshop participants is being implemented • Opinions with respect to progress in implementing new practices varied: o Difficulties cited but positive results observed; o Trainings partially useful but poor implementation.	• Lack of funds to hold workshop and financially support those interested in attending.	• Although no facilitators were identified, participants expressed the utility of the workshops and how they would encourage other health care professionals to attend or be involved in offering future workshops.	• Most participants acknowledged the need to send additional staff to obtain guideline training around development and evaluation, but there are no plans to offer further workshops. • Participation in more frequent workshops could encourage guideline implementation.
#8: Consider engaging some of the local clinicians on the WHO guidelines development group.	• Some participants cited that no progress has been made, whereas others indicated that preliminary stages of selecting topics and working group members are underway.	• Lack of funds to compensate working group members.	• No facilitators identified by participants.	• Suggestions for engagement: o Use credits if relicensing system is introduced; o Have WHO headquarters send out invitations to join working groups; o WHO could support members for events like academic conferences and training with financial incentives (10 or 20 euros/ daily expenses) to encourage training and participation.
#9: Engage those interested in guideline development and implementation from neighbouring countries in the workshop activities and create a ‘virtual’ community of practice to share experiences and avoid duplication of effort.	• Majority of participants have had meetings and/or trainings to share experiences around common contextual issues. Examples of countries engaged: Albania, Macedonia, Bosnia-Herzegovina, and Serbia. • A few networks and telemedicine communities have been formed as a communication tool to connect to regional hospitals in real time and share experiences around guideline development.	• WHO State Membership is needed for WHO to recognize Kosovo as a country office. • Lack of staff/resources to dedicate to activity. • Lack of clear criteria for selecting group members to join working group, MOH makes final decision. • Administrative and bureaucratic delays (e.g., moving paper work through the system).	• No language barrier exists for Kosovo and Albania, thus making it easier to set up and maintain a virtual community of practice to share experiences.	• The MOH and University Hospital in Tirana (Albania) have guidelines and protocols for nurses/midwives and representatives from Albania could be engaged to share their experience with Kosovo. • Create a webpage through United Nations Environment Program and have a virtual community. • Telemedicine can be used as a virtual community.

### Perceived barriers to applying 2012 guideline implementation workshop recommendations

#### Lack of guidelines and protocols

The majority of participants cited the absence of guidelines and protocols as one of the key barriers to the progress of most of the recommended guideline implementation activities (e.g., the inclusion of a monitoring system for guideline adherence, and the creation of motivational strategies to encourage adherence). Participants revealed that guidelines have remained in the development stages and as a result, activities proposed have been negatively impacted. Stakeholders’ lack of awareness and/or knowledge of the clinical guidelines have contributed to the lack of progress made towards the availability of guidelines. In addition, some have suggested that the lack of understanding between the differentiations of guidelines versus protocols have attributed to this situation as well.

*“…we had problems identifying and clarifying what a clinical guideline and what a clinical protocol means.”*

#### Presence of official procedures, formalities, and bureaucracy

Other participants perceived factors such as official procedures, formalities, and bureaucracy as impediments. For example, although a National Council for guideline and protocol development had been established in 2011, it is no longer active, “*awaiting administrative instruction to renew membership of the committee”* and what is more, changes to key positions (i.e., officials) in the Ministry have triggered other issues and delays to the re- instatement of this committee.

*“We have so many frequent changes at the key positions within the Ministry so, one goes, the other comes, and they don’t see it as a priority issue. So most of our time we are just wasting by advocating and informing newcomers about the importance, either for guidelines or for other issues which are within our mandate.”*

As a result, without this committee, participants expressed that steps to carry forth recommended activities such as the creation of working groups with local representative stakeholders (e.g., clinician groups, researchers and WHO) are futile.

*“If the committee on guideline development is not reactivated, then what is the aim of the group to work, then there is nobody to endorse the guidelines, so it [the process] should parallel somehow, the committee to be reactivated, and the working groups to be endorsed so they could start working their job.”*

#### Lack of decision-making capacity

Aside from the availability of guidelines and protocols in place, participants also expressed frustration with their lack of capacity to make any significant official decisions to support guideline implementation activities. One participant recounted that “*we are not in a position that as outsiders from the Ministry of Health to take lead on the process,”* a statement which is similarly echoed by others:

*“The only problem with coordination meetings is that we don’t have a kind of decision-making role, we can only advocate and we can only suggest and recommend, actually, and we can of course as stakeholders or even clinicians, share our concerns and our achievements or whatever, but we are not a decision-making body.”*

#### Lack of available resources

Other factors that participants perceived to have influenced the progress of guideline implementation activities included the availability of monetary funds, although the perceptions of its impact varied from participant to participant. Lack of monetary funds was perceived by one participant as the barrier amid recommendations to supply future guideline development workshops and engage local clinicians in the process, stating that their budget was insufficient to financially support another workshop and those interested in attending. Whereas, another participant explained that while there are stakeholders who are interested in contributing to working groups with or without financial compensation, it may be more difficult to engage those less interested but more knowledgeable in this area because “*without financial means they are not willing to dedicate their time*.”

#### Lack of motivation

Lastly, lack of motivation was cited by a few participants as a hindrance in some cases. For instance, currently MOH holds no authority over private practices and there seems to be no clear distinction between private and public practice, therefore, as a result clinicians that associate themselves as practicing in the private sectors are less motivated to engage in processes developed in a public health institution. Furthermore, one participant stated that *“motivation of health workers is not a priority in Ministry of Health approach toward improving health, health system performance”* and as such there is a lack of interest amongst health workers to become engaged in initiatives that foster guideline development and implementation.

### Perceived facilitators to applying 2012 guideline implementation workshop recommendations

#### Accessibility of existing guidelines

Efforts to encourage the use of existing international guidelines (i.e., reliable guidelines of other countries and associations) to develop local protocols have indirectly contributed to the availability of guidelines and thus the progress of recommended activities. By doing so, this has in turn helped to mitigate some of the barriers to the progress of the recommendations associated with lack of guidelines and protocols mentioned above.

#### Incentives

The provision of incentives was suggested by some as a means to motivate and encourage engagement. Several participants believed that by offering monetary incentives, certifications and/or credits local clinicians would become more involved and participate in activities such as guideline development working groups/committees.

### Recommendation-specific barriers and facilitators

Table 2 presents the findings of the process evaluation conducted in 2014 organized by the nine recommendations identified in the original workshop in 2012. For each recommendation, we have presented progress to date, barriers, facilitators, and participants’ proposed future directions. The information provided in Table 2 represents the aggregate perspectives of participants in the FGs and interviews.

Participants reported that substantial progress had been made on three recommendations. First, significant progress has been made in developing the centralized system for data collection across clinical settings (recommendation 1). Although the system has not yet been established, a number of steps have been completed and the initiative has gained leadership buy-in. Specifically, it has been added as a priority to the Ministry’s Sectorial Health Strategy 2014–2020. The Electronic-Based National Health Information System is being piloted in three Regions of Kosovo (Pristina, Prizren and Ferizaj) in all three levels of care. This pilot is a necessary pre-requisite to implementation of the Health Insurance System, planned for 2016. They are currently determining the most important and priority SMART (Smart, Measurable, Attainable, Relevant, Trackable) performance indicators to initiate payment for performance, as next step toward improved quality of care. Pay for performance was meant as motivating factor for improved individual and institutional performance and creating competitive health care environment in terms of higher level and/or better quality of clinical guideline implementation. Second, progress has been made in incorporating standards into clinical practice using a monitoring system for guideline adherence (recommendation 2). These efforts have, however, been made primarily at a local rather than national level; the current goal is to scale up these efforts. Barriers to scaling up these monitoring standards nationally include lack of awareness, resource issues (human and financial), and evaluation challenges. Participants proposed several future directions to address these barriers and facilitators. Third, Kosovo stakeholders have successfully reached out to neighbouring countries to provide mutual support for guideline development and implementation (recommendation 9). Participants identified several future directions for building partnerships with other countries.

Participants indicated that limited progress had been made on four recommendations. Although some workshops had been offered, participants saw a need for additional capacity building and support after the training (recommendation 7). Participants perceived lack of funds for training as the primary barrier to capacity building and believed that there were opportunities for participants to support each other after attending workshops. For the recommendations that involve developing working groups (recommendations 5, 6, and 8), participants thought little progress had been made over the past two years, primarily due to system-level barriers.

Two recommendations describe a cultural shift in perspectives that may enable guideline implementation; however, these recommendations are not as readily actionable as are some of the other recommendations. Specifically, there is a need to create motivational strategies, such as incentives for health care staff, to encourage guideline adherence and enhance communication across stakeholder groups, including clinicians, managers, and policymakers through participation in such activities as guideline development committees (recommendations 3 and 4). Stakeholders made less progress in both of these areas and identified several barriers that may have prevented progress.

## Discussion

### Updated recommendations

In October 2012, we conducted activities to plan the process of implementing the WHO guideline on the prevention and treatment of PPH in Kosovo. Nine key recommendations and a supporting report on findings were developed as a result of the activities [11]. Two years after the workshop, we completed a realist process evaluation using FGs and key informant interviews to assess participants’ perspectives on progress on the nine recommendations to date, barriers and facilitators experienced related to implementing each recommendation, and possible future directions. The realist approach to the process evaluation allowed us to understand how the characteristics of the implementation setting affected the outputs and progress reported by participants in relation to operationalization of the nine recommendations [15]. One of the lessons learned from the first workshop in Kosovo, and from subsequent similar activities conducted in other LMICs [18–20], was to develop concrete and actionable recommendations for implementation that are grounded in behaviour change theory [21]. Therefore, we developed five updated recommendations: a) develop a guideline development/adaptations working group to create or adapt maternal and perinatal health guidelines; b) develop a guideline implementation working group to pilot test an evidence-informed implementation strategy to support guideline implementation, then scale up the initiative; c) develop guideline policy working groups to work collaboratively with MOH and clinician stakeholders to identify and support opportunities to engage in policy changes related to guideline implementation; d) continue to develop and operationalize a centralized system for data collection; and e) expand guideline development and implementation capacity building and networking activities. Table 3 presents five recommendations adapted from the original recommendations based on progress to date, barriers and facilitators, and suggestions made by participants for improvement. To ground the work in theory, we have also presented elements of the behaviour change wheel [21] that are relevant to each stakeholder group.

**Table 3:** New recommendations proposed by study team

New proposed recommendation	Relevant recommendations from 2012	Possible next steps to operationalize recommendation	Policy and intervention functions addressed by activities
A) Develop a guideline development/adaptations working group to create or adapt maternal and perinatal health guidelines.	#8: Consider engaging some of the local clinicians on the WHO guidelines development group.	*Guideline for critical appraisal:* • Prioritize a WHO guideline to critically appraise within the local context (for example using AGREE II). • Could link with data collection group to identify topics with higher needs (e.g., PPH). • Liaise with people who have been trained in critical appraisal or complete online AGREE training (see recommendation C). *Guideline adaptation:* • Identify whether the guideline needs to be adapted to the local context. • Liaise with the people who have been trained in guideline adaptation (see recommendation E). • Adapt guideline to local context using ADAPTE process.	*Policy functions:* • Guidelines • Regulation
B) Develop a guideline implementation working group to pilot test an evidence-informed implementation strategy to support guideline implementation, then scale up the initiative.	#5: Create a guideline implementation working group with representative stakeholders at the local level. #2: Incorporate standards into clinical practice including a monitoring system for guideline adherence.	*Explore networking opportunities:* • Work with stakeholders who have started implementation activities locally or in other countries. • Liaise with those developing centralized data systems to use data whenever possible in the evaluation (see recommendation D). • Invite members from the new division in the MOH focusing on monitoring and evaluating health services. *Secure resources:* • Apply for seed grant funding from GREAT Network in Fall 2015 building on existing initiatives. • Seek funding to scale up the project. *Capacity-building:* • Send 1–2 people to a KT practice course to learn about how to apply the science of implementation. *Start small and scale up:* • Implement and evaluate a pilot project – prioritize initiatives that emphasize increased communication and motivation (themes 1 and 2).	*Pilot project could address some or all of the following intervention functions:* • Education • Training • Persuasion • Modeling • Enablement
C) Develop guideline policy working group to work collaboratively with the MOH and clinician stakeholders to identify and support opportunities to engage in policy changes related to guideline implementation.	#6: Develop a small working group with local representatives from clinician groups, the MOH guidelines committee and quality portfolio, clinical or health services researchers, and the WHO to move forward with implementation.	• Work on building strong relationships across stakeholder groups (recommendation A). • Maximize opportunities to involve outsiders in work (e.g., MOH on clinician-led initiatives and clinicians on MOH-led initiatives). • Work with MOH to identify opportunities to embed guideline implementation changes at a policy level (e.g., through the new monitoring and evaluation division of MOH and the national level performance based incentive program). • Prepare evidence briefs on guidelines for policymakers. • Use KT Platform model examples from other countries such as Malawi and Uganda.	*Policy functions:* • Regulation • Fiscal measures • Legislation • Communications/marketing *Intervention functions:* • Environmental restructuring
D) Continue to develop and operationalize a centralized system for data collection.	#1: Create a centralized system for data collection across clinical settings as well as for formal and informal channels for practice sharing.	• Continue with efforts to develop a centralized data collection system. • Create a working group to identify key success factors in locally driven initiatives. • Connect with other working groups to support guideline development and implementation efforts.	*Policy functions:* • Environmental/social planning • Regulation *Intervention functions:* • Environmental restructuring
E) Expand guideline development and implementation capacity building and networking activities.	#7: Consider offering workshops on guideline development methods, including use of GRADE (Guyatt et al., 2008), on appraisal of guidelines using AGREE, and on guidelines adaptation (National Collaborating Centre for Methods and Tools [NCCMT], 2011), for representatives from the MOH and clinical groups. #9: Engage those interested in guideline development and implementation from neighbouring countries in the workshop activities and create a ‘virtual’ community of practice to share experiences and avoid duplication of effort.	• Build a capacity building working group or community of practice. • Liaise with other working groups to continue to build capacity and support ongoing activities. • Find education/training opportunities including: o AGREE II tool - critical appraisals o ADAPTE process and toolkit – guideline adaptation o Systematic reviews o Pragmatic trials • KT practice/implementation to develop a train-the-trainer model, where those who have been trained have an opportunity to share what they learned locally. • Use implementation coaching to provide technical support during ongoing implementation efforts.	*Intervention functions:* • Education • Training • Enablement

These recommendations reflect the planning phase of implementation, preparing the system and organizations for guideline implementation, also referred to as readiness, "the extent to which organizational members are both psychologically and behaviorally prepared to implement change" [22]. Although groups may wish to jump into implementation, process models explicitly describe readiness as a step in implementing evidence in practice [12, 23, 24], given that readiness predicts implementation and outcomes [25–27]. In addition to understanding organizational and individual readiness, in LMICs there is a need to also focus on system readiness [26]. Infrastructure (or lack of infrastructure) can facilitate or hinder implementation and outcomes; therefore building a strong infrastructure can have long-term benefits [28, 29]. There are tools to address system readiness in LMICs which can help in this process, such as the Intervention and Research Readiness Engagement and Assessment of Community Health Care (I-RREACH) [30].

### Cross-cutting principles

In reviewing the progress to date on the recommendations, we noted that two of the recommendations involve more of a cultural/system-level shift and are, therefore, more difficult to operationalize. Specifically, the in-country work in 2012 identified two significant barriers to implementation: lack of communication between key stakeholder groups and a focus on punitive interventions (rather than taking a more positive, motivational approach) [11]. Both of these barriers are likely influenced by the socio-political climate in Kosovo since the disintegration of Yugoslavia [31], and therefore are not likely to change quickly, nor are they entirely within the sphere of influence of the key stakeholders involved in guideline implementation. It is important to consider the multi-faceted and interactive effects of personal and environmental factors that determine behaviours [32]. Given this context, we have added two cross-cutting principles that all working groups should consider when undertaking guideline development and implementation work:

1. Establish a mechanism for continuous communication, collaboration, and experience-sharing between key stakeholder groups (e.g., maternal and child health forum); and
2. Foster teamwork culture and collective responsibility rather than focusing on individualization and personalization; this shift could focus on building motivation and using incentivized strategies, rather than focusing on punishment.

### Application in other contexts

The findings of this process evaluation are transferrable to other areas of maternal health work and implementation of evidence-based guidelines, particularly in LMIC settings. Many of the barriers and facilitators experienced by health care system stakeholders in Kosovo also exist in other contexts [33], and the need for specific and actionable recommendations to operationalize implementation efforts is crucial. Recommendations should clearly define the “who”, “what”, “when”, and “how” of an implementation strategy so that stakeholders understand their roles, the objectives, the mechanisms of change, and the anticipated outcomes.

Enabling systems change is challenging [34], and the addition of system-level conflict and instability, which are a reality in many LMIC contexts, exacerbates these issues. Given this context, it is imperative to build strategic partnerships across sectors and levels of government, advocating for policy change, and building the capacity of health care providers and other health care system stakeholders. Existing KT platforms that provide guidance and support on policy engagement can help support these efforts, such as EVIPNet and the Supporting Use of Research Evidence (SURE) [35]. These can be particularly helpful in thinking through how best to communicate with stakeholders (e.g., policymakers) and how to rapidly respond to policy requests [36, 37, 3]. Additionally, ongoing implementation and technical support for working groups in LMICs (i.e., training and coaching) can be provided by groups like the GREAT Network to aid in-country stakeholders in achieving and sustaining implementation goals [38].

### Limitations

Given that research and implementation activities largely stem from time-limited grants, little research is done to evaluate the long-term activities and impacts of implementation efforts. Therefore, this study presents a unique perspective on implementation activities by following-up on activities two years after the initial workshop. However, some limitations of the study should be noted. Although we were able to collect data from a large number of participants, the size of the focus group was larger than ideal [39]. We had the opportunity to include a wide range of stakeholders in the focus group by combining the focus group with an existing meeting; however, this resulted in a large number of participants, all of whom may not have been able to express their views. Consequently, we decided to conduct interviews with a few key stakeholders to make sure that we captured their perspectives. In addition, communication challenges between groups were identified as a major barrier in 2012 and continue to pose challenges. We had limited MOH representation and involvement. Ideally, we wanted to collect data from the MOH perspective as they are a driving force or barrier behind many of the activities but they declined participation. Finally, our understanding and interpretation of the data were limited by cultural barriers and local contextual factors. To reduce the impact of this limitation, an in-country expert was consulted throughout the process to enhance comprehension of the data and its relevance to the local context; this individual is included as a co-author on this paper.

## Conclusion

Using a realist evaluation approach, we assessed activities and progress over a two-year period on the nine recommendations designed to support Kosovo in implementing the WHO guideline on the prevention and treatment of PPH. The evaluation findings provided rich insights into the barriers and opportunities experienced to date. They have also led to the development of five additional recommendations, which were adapted from the original recommendations that can be operationalized by health care system stakeholders in Kosovo. While developed for implementation in Kosovo, these recommendations are transferrable and can be adapted and applied in other LMIC settings because the barriers and facilitators to implementation are shared [33]. Reporting on process evaluation findings, particularly in the context of implementation initiatives, is important for providing insight into realistic targets and results for changes in behavioural outcomes. It is beneficial to share these lessons learned throughout the implementation process for use in other settings. Next steps will involve evaluating how operationalizing the five recommendations has affected outcomes related to use of the WHO prevention and treatment of PPH guideline in Kosovo. Once data monitoring systems are established, future evaluations will examine the degree of WHO maternal health guideline implementation.

## Additional files

Additional file 1: Interview guide. (ZIP 54 kb) Additional file 2: Workshop summary document. (ZIP 42 kb)

## Abbreviations

FG: Focus group
GRADE: Grades of recommendation assessment development and evaluation
GREAT: Guideline development research priorities, evidence synthesis, applicability of evidence, and transfer of knowledge
HRP: Special program of research development and research training in human reproduction
KT: Knowledge translation
LMIC: Low- or middle-income country
MOH: Ministry of Health
PPH: Postpartum haemorrhage
SMH: St. Michael’s Hospital
UNDP: United Nations Development Programme
UNFPA: United Nations Population Fund
UNICEF: United Nations Children’s Fund
WHO: World Health Organization

## Footnotes

1 The first cause are infections (8 out of 24 or 33%).
