# Supplementary material for: Navigating barriers: two-year follow up on recommendations to improve the use of maternal health guidelines in Kosovo
Source: BMC Public Health. 2016 Sep 15;16:987. doi: 10.1186/s12889-016-3641-5 (PMC5025590; doi:10.1186/s12889-016-3641-5)
Supplement: Additional file 1: — Interview guide. (ZIP 54 kb) [file 12889_2016_3641_MOESM1_ESM.zip › Additional File 1_Focus Group GuideR3.docx]

| **Phase 2: Follow-Up Focus Group Guide for GREAT Project Participants** | |
| --- | --- |
| **Instructions for facilitators:**   - *Welcome and introductions* - *Collect signed consent forms* - *Review process for focus group discussion* - *Direct participants to review the summary sheet of barriers and recommendations/worksheet provided. Note: there is a column included in the summary sheet which is intended for participants to write down any key points related to barriers and/or recommendations that they would like to keep confidential or in the event that time does not permit them to share during the session. The worksheets are to be collected by the facilitator at the end of the session.* - *The questions included in this guide are meant to generate open discussion* | |
| **Questions for participants**  **Legend:**   - **Questions** and *Instructions* are indicated as such in the left hand column. *Instructions* are meant to be directions for the participants, given to them by the facilitator. - Directions for the facilitator are indicated in *italics* in the body of the text of the second column. | |
|  | *Instructions*  As you may recall, in October 2012, a two-day meeting (Phase 1) was held with local stakeholders in Kosovo and we would like to evaluate progress made on guideline implementation activities to date.  For Phase 2 of the study, the project team will conduct *focus group discussions* in order to document and assess progress made on implementation activities and strategies since the in-person meeting in October 2012.  We would like to explore your perceptions on the implementation of activities that have occurred since the meeting. The information gathered today will inform a publication documenting chronologically the activities and strategies implemented to date as related to relevant evidence-based guidelines. |
| **Question 1** | *Instructions*  Following the outcomes of the focus groups and consensus meeting held in October 2012, a number of recommendations were made in the publication entitled ‘**Determinants of implementation of maternal health guidelines in Kosovo: Mixed methods study*’.*** We would now like to hear about the progress you have made on the key activities outlined in these recommendations. If you are not aware of or familiar with the activities of a particular recommendation, you may skip over the questions specific to that recommendation.  **Recommendation #1**: Create a centralized system for data collection across clinical settings as well as for formal and informal channels for practice sharing.   - What steps have been taken to create a centralized system for data collection? Please describe. - What steps have been taken towards developing and utilizing channels for practice sharing? Please describe. |
| **Question 2** | **Recommendation #2**: Incorporate standards into clinical practice including a monitoring system for guideline adherence.   - What types of standards have you incorporated in your clinical practice to ensure guideline adherence? - Is a monitoring system being used? Please describe. - How effective have these standards been? Why or why not? |
| **Question 3** | **Recommendation #3**: Create motivational strategies such as, incentives for health care staff, (including managers and clinicians) to encourage guideline adherence.   - Have you utilized motivational strategies to encourage guideline adherence? - If so, please provide examples. - If not, are there plans to utilize motivational strategies in the future? - Have these strategies been effective in encouraging guideline adherence? Why or Why not? |
| **Question 4** | **Recommendation #4**: Increase communication across stakeholder groups including clinicians, managers and policy makers through participation in activities such as guideline development committees.   - Have guideline development committees been formed? - Have stakeholder groups participated in these committees? - Are there other ways communication may be increased across stakeholder groups? |
| **Question 5** | **Recommendation #5**: Create a guideline implementation working group with representative stakeholders at the local level.   - Has a guideline implementation working group been created? - Who are the members of this group and what are their roles? |
| **Question 6** | **Recommendation #6**: Develop a small working group with local representatives from clinician groups, the Ministry of Health guidelines committee and quality portfolio, clinical or health services researchers, and the WHO to move forward with implementation.   - Has this working group with representation from each of these groups (clinician groups, the Ministry of Health guidelines committee and quality portfolio, clinical or health services researchers, and the WHO) been created? - What are some of the activities this working group has been involved with? Please describe with examples. |
| **Question 7** | **Recommendation #7**: Consider offering workshops on guideline development methods (including use of GRADE (Guyatt et al., 2008), on appraisal of guidelines using AGREE, and on guideline adaptation (National Collaborating Centre for Methods and Tools [NCCMT], 2011), for representatives from the Ministry of Health and clinical groups.   - Have these types of workshops been offered? If so, how useful have these been? - Are there plans for offering these types of workshops in the future? |
| **Question 8** | **Recommendation #8**: Consider engaging some of the local clinicians on the WHO guidelines development group.   - Have local clinicians been engaged? Why or Why not? - What are some ways in which local clinicians can be further engaged? |
| **Question 9** | **Recommendation #9**: Engage those interested in guideline development and implementation from neighbouring countries in the workshop activities and create a ‘virtual’ community of practice to share experiences and avoid duplication of effort.   - Have neighbouring country representatives been engaged? - Do opportunities exist for creating a ‘virtual’ community of information and practice sharing? Please describe. |
| **Question 10** | Please describe your thoughts regarding the 2-day meeting held in Kosovo in October 2012 to identify priorities, barriers, and facilitators related to the implementation of WHO maternal guidelines.   - What did you find useful about the 2-day meeting? - What do you think could be done to make the workshop more effective? - Do you feel confident in your knowledge to practice these recommendations? In your skills? In your ability? - Do you think you will require additional training to perform these tasks as recommended by the WHO? |
| **Question 11** | Do you have any additional suggestions that could help with the implementation of the selected WHO guidelines in Kosovo?   - Is there anything else that you would like to add? |
| **Thank participants and wrap up**  **Collect summary/worksheets from participants** | |
