# Supplementary material for: Navigating barriers: two-year follow up on recommendations to improve the use of maternal health guidelines in Kosovo
Source: BMC Public Health. 2016 Sep 15;16:987. doi: 10.1186/s12889-016-3641-5 (PMC5025590; doi:10.1186/s12889-016-3641-5)
Supplement: Additional file 1: — Interview guide. (ZIP 54 kb) [file 12889_2016_3641_MOESM1_ESM.zip › Additional File 2_Interview GuideR3.docx]

| **Phase 2: Follow-Up Interview Guide for GREAT Project Participants** | |
| --- | --- |
| **Instructions for facilitators:**   - *Welcome and introductions* - *Collect signed consent form* - *Review process for interview* | |
| **Questions for participants**  **Legend:**   - **Questions** and *Instructions* are indicated as such in the left hand column. *Instructions* are meant to be directions for the participants, given to them by the facilitator. - Directions for the facilitator are indicated in *italics* in the body of the text of the second column. | |
|  | *Instructions*  As you may recall, in October 2012, a two-day meeting (Phase 1) was held with local stakeholders in Kosovo and we would like to evaluate progress made on guideline implementation activities to date.  For Phase 2 of the study, the project team will conduct individual interviews in order to document and assess progress made on implementation activities and strategies since the in-person meeting in October 2012.  We would like to explore your perceptions on the implementation of activities that have occurred since the meeting. The information gathered today will inform a publication documenting chronologically the activities and strategies implemented to date as related to relevant evidence-based guidelines. |
| **Question 1** | *Instructions*  Following the outcomes of the focus groups and consensus meeting held in October 2012, a number of recommendations were made in the Kosovo report entitled ‘GREAT (**G**uideline-drive, **R**esearch priorities, **E**vidence synthesis, **A**pplication of evidence, and **T**ransfer of knowledge) Interim Project Report: ***Findings from Focus Groups and Consensus Meeting in Pristina, Kosovo’.*** We would now like to hear about the progress you have made on the key activities outlined in these recommendations. If you are not aware of or familiar with the activities of a particular recommendation, you may skip over the questions specific to that recommendation.  **Recommendation #1**: Create a centralized system for data collection across clinical settings as well as for formal and informal channels for practice sharing.   - What steps have been taken to create a centralized system for data collection? Please describe. - What steps have been taken towards developing and utilizing channels for practice sharing? Please describe. - Do you think that these steps have been useful? Why or Why not? |
| **Question 2** | **Recommendation #2**: Incorporate standards into clinical practice including a monitoring system for guideline adherence.   - What types of standards have you incorporated in your clinical practice to ensure guideline adherence? - Is a monitoring system being used? Please describe. - In your opinion, have these standards been effective? Why or why not? |
| **Question 3** | **Recommendation #3**: Create motivational strategies such as, incentives for health care staff, (including managers and clinicians) to encourage guideline adherence.   - Have you utilized motivational strategies to encourage guideline adherence? - If so, please provide examples. - If not, are there plans to utilize motivational strategies in the future? - Have these strategies been effective in encouraging guideline adherence? Why or Why not? - In your opinion, what other strategies could be used to increase guideline adherence? |
| **Question 4** | **Recommendation #4**: Increase communication across stakeholder groups including clinicians, managers and policy makers through participation in activities such as guideline development committees.   - Have guideline development committees been formed? Please describe (e.g., structure, number of members, meeting frequency, etc.) - Have stakeholder groups participated in these committees?   - If so, which ones?   - If not, what have been the barriers to stakeholder engagement? - In your opinion, are there other ways communication may be increased across stakeholder groups? |
| **Question 5** | **Recommendation #5**: Create a guideline implementation working group with representative stakeholders at the local level.   - Has a guideline implementation working group been created? - If so, who are the members of this group and what are their roles? - Please describe some of the activities of this group. - If not, are there plans to create a working group in the future? |
| **Question 6** | **Recommendation #6**: Develop a small working group with local representatives from clinician groups, the Ministry of Health guidelines committee and quality portfolio, clinical or health services researchers, and the WHO to move forward with implementation.   - Has this working group with representation from each of these groups (clinician groups, the Ministry of Health guidelines committee and quality portfolio, clinical or health services researchers, and the WHO) been created? - What are some of the activities this working group has been involved with? Please describe with examples. |
| **Question 7** | **Recommendation #7**: Consider offering workshops on guideline development methods (including use of GRADE (Guyatt et al., 2008), on appraisal of guidelines using AGREE, and on guideline adaptation (National Collaborating Centre for Methods and Tools [NCCMT], 2011), for representatives from the Ministry of Health and clinical groups.   - Please describe the content and format of the AGREE/ADAPTE workshop that was offered in December 2013. - In your opinion, how useful have these workshops been? - Are there plans for offering these types of workshops in the future? |
| **Question 8** | **Recommendation #8**: Consider engaging some of the local clinicians on the WHO guidelines development group.   - Have local clinicians been engaged? How have they been engaged? - What are some ways in which local clinicians can be further engaged? |
| **Question 9** | **Recommendation #9**: Engage those interested in guideline development and implementation from neighbouring countries in the workshop activities and create a ‘virtual’ community of practice to share experiences and avoid duplication of effort.   - Have neighbouring country representatives been engaged? - If so, how have they been engaged? - If not, what were some of the barriers to engagement? What are some strategies which may be used to engage them? - Do opportunities exist for creating a ‘virtual’ community of information and practice sharing? Please describe. |
| **Question 10** | Please describe your thoughts regarding the 2-day meeting held in Kosovo in October 2012 to identify priorities, barriers, and facilitators related to the implementation of WHO maternal guidelines.   - What did you find useful about the 2-day meeting? - What do you think could be done to make the workshop more effective? - Do you feel confident in your knowledge to practice these recommendations? In your skills? In your ability? - Do you think you will require additional training to perform these tasks as recommended by the WHO? |
| **Question 11** | Do you have any additional suggestions that could help with the implementation of the selected WHO guidelines in Kosovo?   - Is there anything else that you would like to add? - Is there anything on your completed summary sheet you would like to highlight? |
| **Thank participants and wrap up**  **Collect summary/worksheets from participant** | |
