# Supplementary material for: Navigating barriers: two-year follow up on recommendations to improve the use of maternal health guidelines in Kosovo
Source: BMC Public Health. 2016 Sep 15;16:987. doi: 10.1186/s12889-016-3641-5 (PMC5025590; doi:10.1186/s12889-016-3641-5)
Supplement: Additional file 2: — Workshop summary document. (ZIP 42 kb) [file 12889_2016_3641_MOESM2_ESM.zip › Additional File 4_Summary Document for InterviewsR3.docx]

Background

A focus group and one-day meeting was held in Pristina, Kosovo in October 2012 in which local stakeholders discussed the local barriers and facilitators to the implementation of the WHO guidelines in diverse clinical settings across Kosovo (Phase 1).

For Phase 2 of the study, the project team will conduct interviews and focus group discussions with the local stakeholders in order to document and assess progress made on implementation activities and strategies since the in-person meeting in October 2012.

Instructions

In preparation of these activities we would request that you share your perceptions on implementation of activities to date. Please use this worksheet to record key points related to the barriers/recommendations that were identified in the meeting in 2012. *Please return this sheet to the facilitator at the end of the interview.*

**Part 1: Barriers to Implementation**

During the focus groups, participants discussed the local barriers to the implementation of the WHO guidelines in diverse clinical settings across Kosovo. Please use the column on the right to indicate any activities that have taken place to address each identified barrier:

|  | **Barriers Identified** | **Related activities to address the identified barrier** |
| --- | --- | --- |
| Barriers at the Health Care Provider Level | Lack of understanding of and engagement in WHO guideline development process |  |
|  | Lack of capacity to document and monitor clinical practice |  |
|  | Lack of communication and agreement among clinician groups (specifically, obstetricians and midwives) |  |
|  | Lack of training and continuing education opportunities available for clinicians and undergraduate students |  |
| Barriers at the Health Care Systems Level | Fragmented system (post-conflict) |  |
|  | Lack of communication between Ministry of Health and clinician organizations |  |
|  | Lack of clarity regarding roles and responsibilities of various clinicians, managers and policy makers |  |
|  | Lack of a centralized system to document and access clinical data |  |
|  | Lack of resources to implement the guideline such as relevant medications and equipment |  |

**Part 2: Recommendations**

Following the outcomes of the focus groups and consensus meeting held in October 2012, a number of recommendations were made in the publication entitled ‘**Determinants of implementation of maternal health guidelines in Kosovo: Mixed methods study*’****.* Please use the column on the right to indicate any activities that have taken place in relation to these recommendations:

| **Recommendations** | **Related activities in line with the recommendation** |
| --- | --- |
| 1. Create a centralized system for data collection across clinical settings as well as for formal and informal channels for practice sharing. |  |
| 1. Incorporate standards into clinical practice including a monitoring system for guideline adherence. |  |
| 1. Create motivational strategies such as, incentives for health care staff, (including managers and clinicians) to encourage guideline adherence. |  |
| 1. Increase communication across stakeholder groups including clinicians, managers and policy makers through participation in activities such as guideline development committees. |  |
| 1. Create a guideline implementation working group with representative stakeholders at the local level. |  |
| 1. Develop a small working group with local representatives from clinician groups, the Ministry of Health guidelines committee and quality portfolio, clinical or health services researchers, and the WHO to move forward with implementation. |  |
| 1. Consider offering workshops on guideline development methods (including use of GRADE (Guyatt et al., 2008), on appraisal of guidelines using AGREE, and on guideline adaptation for representatives from the Ministry of Health and clinical groups. |  |
| 1. Consider engaging some of the local clinicians on the WHO guidelines development group. |  |
| 1. Engage those interested in guideline development and implementation from neighbouring countries in the workshop activities and create a ‘virtual’ community of practice to share experiences and avoid duplication of effort. |  |
